# A Case of Strangulated Inguinal Hernia, in an Inmate of the State Lunatic Asylum

**Published:** 1887-10

**Authors:** A. N. Denton

**Affiliations:** Austin; Late Superintendent, President Travis County Medical Society


### A CASE OF STRANGULATED INGUINAL HERNIA IN AN INMATE OF THE STATE LUNATIC ASYLUM: OPERATION.

*By A. N. Denton, M. D., Late Superintendent, President Travis County Medical Society.*

Read before Travis County Medical Society, and voted for publication in Daniel's Texas Medical Journal.

ON the evening of January 30, 1886, Mrs. Nannie Smith, aged 42, who, for some years, had been troubled with inguinal hernia (direct), was found to be suffering with strangulation of the same.

The patient was the unfortunate victim of chronic mania, in feeble health, and much emaciated. She had for several years been an inmate of the State Lunatic Asylum. Apparently a more unfavorable case upon which to perform a severe operation could scarcely be imagined.

During the evening of the 30th, repeated but unsuccessful efforts at reduction were made, under the influence of an anæsthetic. Some three hours were thus spent. The hips and lower extremities were elevated, while the reduction by taxis was being attempted. Warm baths were also used, with a view to relaxation, but all to no avail.

At 9 p. m., grs ⅓ of morphia was administered hypodermically, she being extremely restless, and suffering much pain. This was repeated at 10 p. m. Notwithstanding the morphine, she passed a miserable and restless night, suffering with constant nausea, and stercoraceous vomiting. On the morning of the 31st her condition was apparently much worse; the radial pulse could scarcely be felt; the face wore a pinched and haggard expression, indicative of the near approach of dissolution; the extremities were cold, and the surface was bathed in perspiration.

Another unsuccessful effort, by traxis, was made at reduction, and, although the patient appeared to be in a dying condition, it was decided to operate, and release the constricted bowel. Accordingly, at 11 a. m., assisted by Dr. F. A. Maxwell, who administered a few whifs of ether, I proceeded to operate in the usual manner. I may sta[illegible]e, in passing, that life was apparently so nearly extinct that it was deemed unnecessary to administer any considerable quantity of the anæsthetic. The incisions being completed, the internal ring was felt for with the index finger of the left hand, which served as a guide, protecting the constricted gut beneath, and finding it, it was divided with a hernia knife, when the hernia was reduced without further trouble, and without opening the sac. The wound was closed with interrupted sutures, and adhesive strips and a compress and bandage applied.

In half an hour after the completion of the operation, the pulse grew stronger, and respiration was more tranquil and less superficial. At 3 p. m., the patient had fairly reacted, and her pulse became regular, but was still feeble; the extremities became warmer, and, with the exception of some nausea which still remained, she was seemingly doing well.

[The daily record of the case is omitted.]

February 4. Pulse 84. There was some inflammation at the seat of the wound, but it had a healthy appearance. From this date her recovery was steady and without interruption, until the wound was entirely healed; and the patient was restored to her usual condition of health.

This case is reported, not because of anything new, or because of any peculiar merit in the operation, or management of the case, but to illustrate the value of surgical measures, as a *derneir resort* in cases that are apparently hopeless; as well as to encourage the surgeon when all else fails, to operate, even though there is scarcely a glimmer of hope, and death is apparently about to claim his victim. In this case, no one present believed that the patient would survive the operation; but, notwithstanding her desperate condition, she made a good recovery, and to-day is in the enjoyment of a fair degree of physical health.

